# About the need to use specific population references in estimating paediatric hypertension: Sardinian blood pressure standards (age 11-14 years)

**DOI:** 10.1186/1824-7288-38-1

**Published:** 2012-01-10

**Authors:** Pier Paolo Bassareo, Andrea Raffaele Marras, Giuseppe Mercuro

**Affiliations:** 1 Department of Cardiovascular and Neurological Sciences, University of Cagliari, Italy; 2 Study Center for Cardiac Disease in Paediatric Age, University of Cagliari, Italy

## Abstract

**Background:**

Previous Italian paediatric blood pressure (BP) tables overestimated the prevalence of hypertension in adolescents of specific geographic areas, such as Sardinia, an island in the Mediterranean Sea. This is probably due to a not very homogeneous distribution of the subjects studied, most from Middle and Northern Italy, and the long period from the survey.

**Methods:**

BPs were repeatedly measured over a period of 3 years in 839 children (52.6% males. Age range: from 11 to 14 years during this period), using a standard mercury sphygmomanometer. For each gender, the specific percentile curves of systolic and diastolic BP were constructed.

**Results (corrected by the 50^th ^percentile of height):**

**Males (11-14 years)**

mean systolic BP (50^th ^centile): from 111 to 115 mmHg. Hypertensive systolic BP (> 95^th ^percentile): from 127 to 135 mmHg. Mean diastolic BP (50^th ^centile): from 65 to 69 mmHg. Hypertensive diastolic BP (> 95^th ^percentile): from 78 to 82 mmHg.

**Females(11-14 years)**

mean systolic BP (50^th ^centile): from 110 to 112 mmHg. Hypertensive systolic BP (> 95^th ^percentile): from 127 to 130 mmHg. Mean diastolic BP (50^th ^centile): from 65 to 67. Hypertensive diastolic BP (> 95^th ^percentile): from 78 to 80 mmHg.

**Conclusions:**

Sardinian BP tables emphasizes the need to integrate the previous standards with more up-to-date and representative reports on Italian children, as periodically performed in the USA, in order to increase the number of subjects to be checked, and to obtain a national coverage better and more completely representative of every geographic area of our country.

## Introduction

At this time, about 30% of all the world adult population is suffering from hypertension (HTN), an important risk factor in the subsequent developing of a number of cardiovascular (CV) diseases, such as stroke or heart attack. Furthermore, this percentage is probably underestimated, because most people do not check their blood pressure (BP) levels regularly and carefully [1].

HTN can be identified since infancy or adolescence, as a fleeting excess in BP values or an anomalous BP rising when anxious or during physical exercise. As for other chronic diseases, HTN can be detected many decades before its signs and symptoms clinically manifested themselves. In addition, since BP tends to track along the same percentiles throughout life, children with higher BPs are more likely to become hypertensive adults [2-4].

It is noteworthy that normal BP values in paediatric age are really different from those in adulthood. So, a Task Force was instituted by the American Academy of Pediatrics in 1977 to develop normative data for BPs in children and adolescents. Updated versions were issued in 1987, 1996, and 2004 [5-7]. Anyway, when using the normative tables established by the United States Task Force, the prevalence of paediatric HTN in other countries or geographic areas is often overestimated [8,9].

In this respect, in 1999 Menghetti et al. developed a national standard level of BP for Italian children, having screened a large sample of the population [10]. Anyway, even when using these more focused references, important differences may arise in estimating HTN in some specific Italian geographic areas. For example, we found isolated diastolic HTN to be most prevalent in Sardinia when using our own curves, and isolated systolic HTN most prevalent when using the tables of Menghetti and colleagues [8].

The aim of this study was to plot the Sardinian normal standards for systolic and diastolic BPs in children aged 11-14 years.

### Materials and methods

The SHARP (Sardinian Hypertensive Adolescent Research Program) study was a longitudinal long term investigation carried out in Sardinia, Southern Italy, to gather information about the prevalence of HTN in children aged 11-14 years. To derive the data, we studied 839 children, of whom 441 (52.6%) were male, attending second-grade schools. Our aim was to investigate the prevalence of HTN by conducting prospective longitudinal assessment of BP over a period of 3 years [8].

The selected subjects were representative of the population of children in both towns and surroundings of Sardinia, a region with a relatively small number of inhabitants. Most were living in Cagliari and in its surroundings [8].

Screening was performed according to a standardized and previously reported recording protocol [11]. All children had their BP measured in their right arm, using a standard mercury sphygmomanometer (F. Bosch Medizintechnik, GmbH & Co. KG, Bisingen, Germany). Measurements were made by two trained physicians after the subjects had rested quietly for at least 10 minutes in a silent classroom. The achievement of a quiet state was checked by measuring heart rate at the wrist.

We made 5 consecutive measurements for each child, recording both systolic and diastolic pressures, and their mean was used for the analysis. A cuff of appropriate size was chosen relative to the circumference of the upper arm, choosing from 2 cuffs with different bladder widths. We used a paediatric cuff for those with circumferences equal to or smaller than 22 centimetres, and an adult cuff for those with circumference exceeding 22 centimetres. In this way, we ensured that the cuff covered two-thirds of the length of the upper arm [12].

The head of stethoscope was placed over the pulse in the brachial artery, proximal and medial to the cubital fossa, which was positioned at the level of the heart, the head of the stethoscope being about 2 centimetres below the bottom edge of the cuff. We used the 1^st ^and 5^th ^phases of the Korotkoff sounds to define systolic and diastolic pressures, respectively. We took care to deflate the cuff slowly, at a rate of 2 millimetres of mercury per second.

Each child in the SHARP study had his/her BP measured every 6 months from February 2005 to June 2007, their ages ranging from 11 to 14 years during this period [8].

### Statistics

For each gender, the specific centile curves of systolic and/or diastolic BP derived from our study were constructed by fitting a third-order polynomial model of BP on age and height using multiple regression analysis.

Subjects in the study were divided into normotensive (10^th^-90^th ^percentile), prehypertensive (91^st^-95^th ^percentile) and hypertensive(> 95^th ^percentile) phenotypes defined by the age-, gender-and height-specific reference standards as presented in the 2004 Working Group charts. In fact, BP is known to be related to age, gender and height in adolescents [7,13,14].

## Results

**Figure 1:**
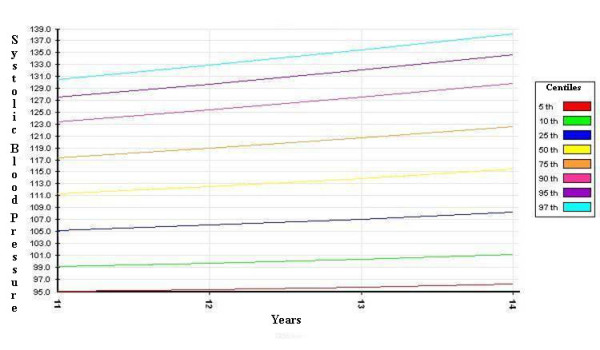
**systolic blood pressure percentiles for boys (corrected by the 50^th ^percentile of height)**.

**Figure 2:**
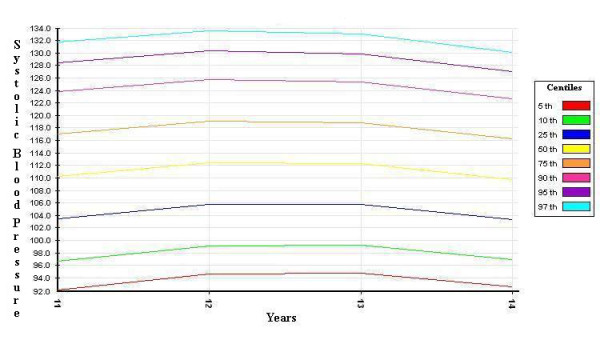
**systolic blood pressure percentiles for girls (corrected by the 50^th ^percentile of height)**.

**Figure 3:**
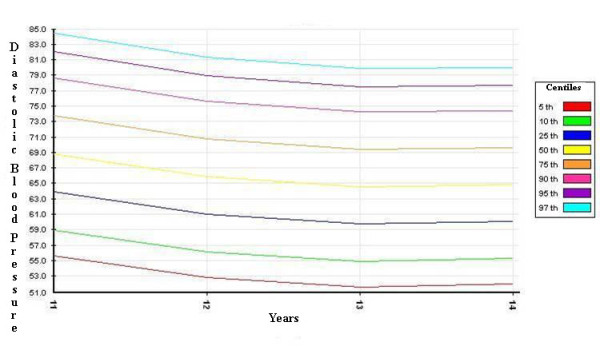
**diastolic blood pressure percentiles for boys (corrected by the 50^th ^percentile of height)**.

**Figure 4:**
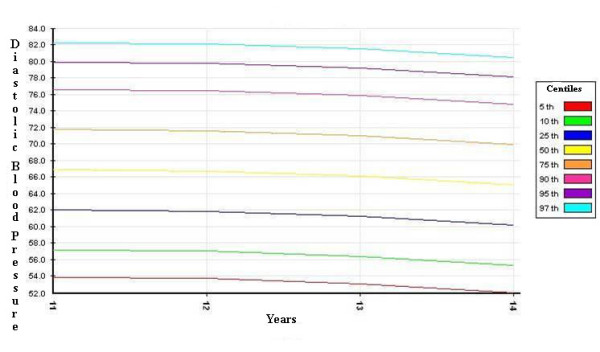
**diastolic blood pressure percentiles for girls (corrected by the 50^th ^percentile of height)**.

The distribution curves of systolic and diastolic BP - derived from the SHARP study, corrected by the 50^th ^percentile of height, and divided by gender - are shown in Figures 1, 2, 3 and 4.

Mean systolic BP values (50^th ^centile) for males were 111, 112, 114, 115 mmHg at 11, 12, 13, and 14 years, respectively.

Hypertensive male subjects for systolic BP, i.e. those having a persistent systolic BP over the 95^th ^centile, were boys whose BP was major than 127, 129, 130, 135 mmHg at 11, 12, 13, and 14 years, respectively.

Mean diastolic BP values (50^th ^centile) for males were 69, 66, 64, 65 mmHg at 11, 12, 13, and 14 years, respectively.

Hypertensive male subjects for diastolic BP (diastolic BP over the 95^th ^centile) were those whose BP was major than 82, 79, 77, 78 mmHg at 11, 12, 13, and 14 years, respectively.

Mean systolic BP values (50^th ^centile) for females, i.e. those having a persistent systolic BP over the 95^th ^centile, were girls whose BP was major than 110, 112, 112, 110 mmHg at 11, 12, 13, and 14 years, respectively.

Hypertensive female subjects for systolic BP (diastolic BP over the 95^th ^centile) were those whose BP was major than 128, 130, 130, 127 at 11, 12, 13, and 14 years, respectively.

Mean diastolic BP values (50^th ^centile) for females were 67, 66, 66, 65 mmHg at 11, 12, 13, and 14 years, respectively.

Hypertensive female subjects for diastolic BP (diastolic BP over the 95^th ^centile) were those whose BP was major than 80, 80, 79, 78 mmHg at 11, 12, 13, and 14 years, respectively.

Statistically significant differences in BP values between genders are absent at 11-12 years (p = ns) and occur later.

The systolic and diastolic BP levels for boys and girls by age and height percentile are shown in Tables 1, 2, 3, and 4.

**Table 1:** Systolic blood pressure levels for boys by percentile of age and height

Years	Percentile of BP	SBP (mmHg) by percentile of height						
		**5^th^**	**10^th^**	**25^th^**	**50^th^**	**75^th^**	**90^th^**	**95^th^**
11	50^th^	106	107	109	111	112	114	114
	90^th^	119	120	121	123	125	126	127
	95^th^	123	124	125	127	129	130	131
12	50^th^	107	108	110	112	114	115	116
	90^th^	120	121	123	125	126	128	128
	95^th^	126	127	129	130	132	134	134
13	50^th^	110	111	112	114	116	117	118
	90^th^	122	123	125	127	129	130	131
	95^th^	127	128	130	132	134	135	136
14	50^th^	110	111	113	115	117	118	119
	90^th^	125	126	128	130	131	133	133
	95^th^	131	132	134	135	137	139	139

Abbreviations: BP: blood pressure; SBP: systolic blood pressure

**Table 2:** Systolic blood pressure levels for girls by percentile of age and height

Years	Percentile of BP	SBP (mmHg) by percentile of height						
		5^th^	10^th^	25^th^	50^th^	75^th^	90^th^	95^th^
11	50^th^	107	108	109	110	112	113	114
	90^th^	121	121	123	124	125	126	127
	95^th^	125	125	126	128	129	130	131
12	50^th^	109	110	111	112	114	115	116
	90^th^	123	123	124	126	127	128	129
	95^th^	126	127	128	130	131	133	133
13	50^th^	109	110	111	112	114	115	115
	90^th^	122	123	124	126	127	128	129
	95^th^	126	127	128	129	131	132	133
14	50^th^	107	107	108	110	111	112	113
	90^th^	121	122	123	124	126	127	127
	95^th^	124	124	126	127	128	130	130

Abbreviations: BP: blood pressure; SBP: systolic blood pressure

**Table 3:** Diastolic blood pressure levels for boys by percentile of age and height

Years	Percentile of BP	SBP (mmHg) by percentile of height						
		5^th^	10^th^	25^th^	50^th^	75^th^	90^th^	95^th^
11	50^th^	67	67	68	69	70	71	71
	90^th^	77	77	78	79	80	81	81
	95^th^	80	80	81	82	83	84	84
12	50^th^	63	64	65	66	67	67	68
	90^th^	74	75	75	76	77	78	79
	95^th^	76	77	78	79	80	80	81
13	50^th^	62	62	63	64	65	66	66
	90^th^	72	72	73	74	75	76	76
	95^th^	76	76	77	78	79	80	80
14	50^th^	62	63	64	65	66	67	67
	90^th^	71	72	73	74	75	75	76
	95^th^	76	76	77	78	79	80	80

Abbreviations: BP: blood pressure; DBP: diastolic blood pressure

**Table 4:** Diastolic blood pressure levels for girls by percentile of age and height

Years	Percentile of BP	SBP (mmHg) by percentile of height						
		5^th^	10^th^	25^th^	50^th^	75^th^	90^th^	95^th^
11	50^th^	66	66	66	67	68	69	69
	90^th^	75	75	75	76	77	78	78
	95^th^	79	79	79	80	81	82	82
12	50^th^	65	65	65	66	67	68	68
	90^th^	75	75	75	76	77	78	78
	95^th^	79	79	79	80	81	81	82
13	50^th^	65	65	65	66	67	68	68
	90^th^	75	75	75	76	77	78	78
	95^th^	78	78	78	79	80	81	81
14	50^th^	64	64	64	65	66	67	67
	90^th^	74	74	74	75	76	77	77
	95^th^	77	77	77	78	79	80	80

Abbreviations: BP: blood pressure; DBP: diastolic blood pressure

Regarding the 90^th ^percentile of BPs, heart rate, height, and weight, a comparison between our paediatric population and USA children is reported in Tables 5, 6, 7 and 8.

**Table 5:** Age-specific 90^th ^percentiles of blood pressure, height, and weight for boys (SHARP study)

	11	12	13	14 years
**Systolic BP (mmHg)**	123	125	127	130
**Diastolic BP (mmHg)**	79	76	74	74
**Height (cm)**	149	151	156	163
**Weight (Kg)**	46	48	51	58

**Table 6:** Age-specific 90^th ^percentiles of blood pressure, height, and weight for boys (USA Task Force)

	11	12	13	14 years
**Systolic BP (mmHg)**	119	121	124	125
**Diastolic BP (mmHg)**	76	77	79	78
**Height (cm)**	153	159	165	172
**Weight (Kg)**	50	55	62	68

**Table 7:** Age-specific 90^th ^percentiles of blood pressure, height, and weight for girls (SHARP study)

	11	12	13	14 years
**Systolic BP (mmHg)**	124	126	126	124
**Diastolic BP (mmHg)**	76	76	76	75
**Height (cm)**	149	151	154	157
**Weight (Kg)**	45	48	48	52

**Table 8:** Age-specific 90^th ^percentiles of blood pressure, height, and weight for girls (USA Task Force)

	11	12	13	14 years
**Systolic BP (mmHg)**	119	122	124	126
**Diastolic BP (mmHg)**	77	78	80	81
**Height (cm)**	154	160	165	168
**Weight (Kg)**	51	58	63	67

## Discussion

It has been reported that despite the availability of charts and electronic programs for normal and abnormal BPs, paediatric clinicians still may find it difficult to integrate such facilities into their routine work flows, thus underestimating the real prevalence of paediatric HTN [15].

Moreover, racial differences in BP patterns in adolescents stimulated the questioning of the validity of using one single diagnostic chart across different racial populations [8,9].

Until now, there have been few studies of the distribution of BPs in a Southern European population of children and adolescents. Indeed, to our knowledge, the SHARP study was the first longitudinal screening of the same subjects performed in Southern Europe, and certainly the first in Italy [8]. Its findings show the need to use population-specific charts when screening for HTN in childhood, and confirm that the tables prepared by the Task Force established in the United States of America overestimate the prevalence of HTN in Italian children [10].

The tables provided by Menghetti et al. in 1999 represented the first important attempt to provide Italian national BP nomograms for children. These standards were prepared using the data available from 21 studies conducted in Italy from 1988 throughout 1994. The use of those age, height and sex-adjusted Italian charts reduces the prevalence of hypertensive children in Sardinia if compared with the numbers derived using the tables prepared in the United States of America [8]. This commendable attempt, however, was less than perfect due to some methodological differences in the form of screening among the various studies, for example the use of isolated versus repeated measurements of BP, a not very homogeneous distribution of the subjects studied, most coming from Middle and Northern Italy, and the long period from the survey.

Our Sardinian study emphasizes the need to integrate these standards with more up-to-date and representative reports on Italian children, as it has been done in the United States of America since 1987, to increase the number of subjects to be checked, and so to obtain better national coverage in order to represent more completely all geographic, socio-economic, and ethnic aspects of the population of our country [8,10].

Through recording BP routine, clinicians from every ethnic group or geographic area in the world should produce their own national/regional nomograms relating to age, gender and height, derived from their genetic, nutritional, cultural, ethnic and social backgrounds. Although the standards established in the United States of America have been adopted worldwide, many local percentile curves are still being used, especially in Northern Europe [16].

In clinical practice, nonetheless, physicians practising in Italy sometimes go on using BP tables derived from USA measurements, rather than the specific population ones [17].

As above stated reported, BP is influenced especially by age, gender and height [7,13,14]. In this respect, we have provided the Sardinian BP reference values according with these three parameters. The same methodological approach had been previously performed by both the USA Task Force on high BP in children and Menghetti [6,7,10]. We have not considered BP levels in relation to weight, because the latter is more correlated to adiposity than to true body size, especially in populations with an elevated prevalence of obesity [10,18]. BP standards based on age, gender and height permit a more precise classification of BP according to body size, avoiding misclassifying children at the extremes of normal growth [6].

A possible pathophysiological explanation of both the lack of statistically significant differences in BP levels between genders at 11-12 years and the BP different trends in our children in comparison with those reported in other studies, may take into account the earlier transition to maturity of our Southern Italian students compared with American or Middle-Northern Italian ones [8]. An early menarche is known to be associated with increased systolic BP, while there is a similar inverse relationship between age at menarche and diastolic BP [19]. The majority of the girls in our study reported their menarche to have occurred no later than when they were 13 years old. A previous study reported both systolic and diastolic pressures increased significantly with age for boys, but not for girls [20]. In the opinion of the Authors, further studies are needed to clarify this interesting issue.

The present study undoubtedly features several limitations: a) a too small number of patients, that should consequently be enlarged in future studies. Anyway, the sample we have investigated was representative of the school-aged children in our island (about 15% of all the people attending secondary school); b) BPs were measured using a standard auscultation protocol instead of an oscillometric device. In fact, it is common knowledge that BP values measured by oscillometric equipments often vary compared with those got from auscultation, so that caution must be exercised in the diagnosis of HTN in children when an automated device is used [21]. Physicians' ability in measuring BP was previously tested by comparing together the BP values each of them have registered in the patients (concordance of 98%). The repeated measurements in the auscultatory mode we have performed also eliminate the influence of the possible observer's bias/human error inherent in the auscultation protocol; c) the need to follow these patients to verify both if the high BP currently recorded in adolescence will really track in adult HTN, and the possible onset of severe CV diseases as time passes (i.e. coronary artery disease, stroke); d) the need to extend the screening to other age ranges.

## Conclusions

CV diseases - such as HTN - are slowly evolving and their pathogenesis often begins in childhood. By routinely using specific population and carefully constructed BP tables, paediatric clinicians and paediatric cardiologists have the possibility to early identify a pathophysiological process that will be clinically manifested only after several decades.

The consequent possibility to prevent BP rising and its dangerous consequences since paediatric age is an attractive perspective in clinical practice, in order to avoid the tracking of a simple early predisposition to a fatal destiny in adulthood.

## Competing interests

The present study was supported by Regione Autonoma della Sardegna - Assessorato alla Sanità with a reimbursement of both fuel costs and statistician's consultation.

## Authors' contributions

PPB: acquisition of data, conception and design - ARM: acquisition of data, revising the manuscript critically - GM: final approval of the version to be published. All authors read and approved the manuscript.
